# Indium *K*α radiation from a MetalJet X-ray source: comparison of the Eiger2 CdTe and Photon III detectors

**DOI:** 10.1107/S1600576723007215

**Published:** 2023-09-05

**Authors:** Paul Niklas Ruth, Nico Graw, Tobias Ernemann, Regine Herbst-Irmer, Dietmar Stalke

**Affiliations:** a Institut für Anorganische Chemie, Georg-August-Universität Göttingen, Tammannstraße 4, Göttingen, Lower Saxony 37077, Germany; Ecole National Supérieure des Mines, Saint-Etienne, France

**Keywords:** In radiation, independent-atom model, multipole model, Hirshfeld atom refinement, MetalJet, detectors

## Abstract

The MetalJet source makes available new *K*α radiation wavelengths for use in X-ray diffraction experiments. The purpose of this paper is to demonstrate the application of indium *K*α radiation in independent-atom model refinement, as well as approaches using aspherical atomic form factors. The results vary greatly depending on the detector employed, as the energy cut-off of the Eiger2 CdTe provides a solution to a unique energy contamination problem of the MetalJet In radiation, which the Photon III detector cannot provide.

## Abstract 2

This paper communicates the first application of MetalJet In *K*α radiation to single-crystal X-ray crystallography and evaluates two different detectors, the Bruker Photon III and the Dectris Eiger2 CdTe, for usage in spherical and aspherical structural models.

## 1. Introduction

In the first set of X-ray diffraction images ever reported, Friedrich, Knipping and Laue used the white beam of commercially available X-ray tubes (Friedrich *et al.*, 1912 ▸). However, only shortly afterwards it was reported that using monochromatic X-ray radiation was beneficial for the interpretability of structures (Bragg & Bragg, 1913 ▸). For single-crystal X-ray diffraction, this has held until today, and the latest and most consequential contribution to this development with in-house instrumentation is the recent report of using copper *K*β (Meurer *et al.*, 2022 ▸) to avoid even the *K*α_1_–*K*α_2_ splitting for the employed X-ray radiation. Sometimes instrumentation has led to unexpected contamination, leading to lower monochromaticity, such as the reported contamination by a higher harmonic of the monochromator, *i.e.* λ/2 contamination (Kirschbaum *et al.*, 1997 ▸), or the lower-wavelength contamination in microfocus sources, which has been eliminated by attenuation (Macchi *et al.*, 2011 ▸) or can be accounted for with an empirical correction (Krause *et al.*, 2015*b* ▸).

In this publication we evaluate the performance of a setup consisting of an Excillum MetalJet D2 source coupled with either a Bruker Photon III or a Dectris Eiger2 CdTe detector. Despite the high-quality Incoatec Helios optics optimized for indium radiation, there is still significant low-energy gallium contamination. Subsequently, we want to compare the traditional approach of suppressing this radiation with an absorber, as required for the Bruker Photon III detector, with the application of the energy cut-off feature of the Dectris Eiger2 CdTe detector, with an additional discussion of other influences on the overall detectable intensity. In order to provide tests representing potential applications of this setup, we want to evaluate the influence of the detector on this specific setup for independent-atom model (IAM) evaluations of reference compounds with both high and low absorption, coupled with aspherical density refinements of the well known YLID crystal (2-dimethylsulfuranylidene-l,3-indanedione) at 110 K.

## 2. Experimental

### 2.1. Investigated structures

**Figure 1:**
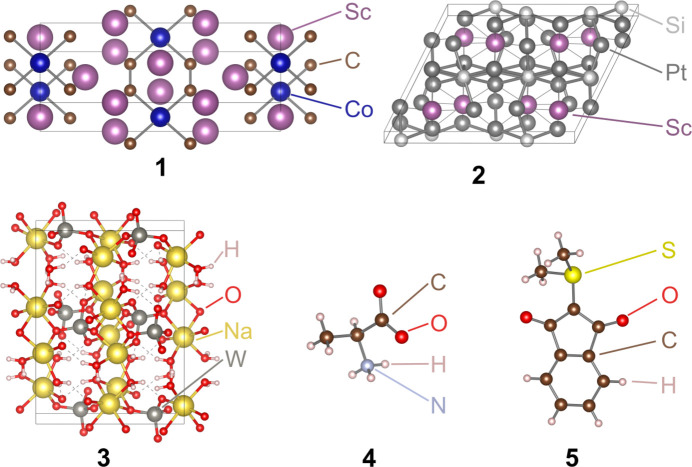
Structures of the compounds **1** to **5**.

Scandium cobalt carbide (Jeitschko *et al.*, 1989 ▸), [ScCoC_4_]_*n*_, **1**, has been the subject of a number of investigations of the solid state (Zhang *et al.*, 2007 ▸; He *et al.*, 2015 ▸), including high-resolution X-ray diffraction studies (Rohrmoser *et al.*, 2007 ▸; Eickerling *et al.*, 2013 ▸; Langmann *et al.*, 2021 ▸), and was therefore chosen together with scandium platinum silicide, [ScPt_9_Si_3_]_*n*_, **2**, and sodium tungstate dihydrate, [Na_2_WO_4_·2H_2_O]_*n*_, **3**, to represent inorganic salts with particularly high absorption coefficients. As a contrast, l-alanine (L-Ala), **4**, was chosen, because it diffracts reasonably well while having a low absorption coefficient. Finally, a C_11_H_10_O_2_S YLID crystal, **5**, was used to compare the two detectors and the setup with a second machine using aspherical refinements. The YLID crystal is an established benchmark for IAM refinements but can also be used for benchmarks at low temperatures (Guzei *et al.*, 2008 ▸), which enables the use of aspherical models. Depictions of the structures within this investigation are shown in Fig. 1 ▸ and crystallographic details are given in Table 1 ▸.

### 2.2. Measurement

The diffraction data with indium radiation were collected on a Bruker D8 Venture four-circle diffractometer with an Excillum MetalJet D2 source using ExAlloy-I3 (75% gallium and 25% indium) and Incoatec Helios optics. With the Bruker Photon III detector, gallium contamination was filtered using a palladium foil of 40 µm thickness. For the Eiger2 CdTe 1M detector, a custom solution within the D8 Venture was implemented. Steering using .exp files as written by Bruker *APEX4* was implemented in Python, while the triggering was implemented using a custom trigger box. As a result, empty Bruker frames containing the goniometer information were collected at the same time as the Eiger2 collected its frames using the same exposure times. Another Python script was used to merge the two frames together. The Eiger2 measurements used no attenuation, but did use an energy cut-off of 12.1 keV to avoid the gallium contamination.

Diffraction data with silver radiation were collected using a Bruker D8 Venture four-circle diffractometer with an Incoatec IμS 3.0 Ag source and a Bruker Photon III detector as is.

### 2.3. Data processing and refinement for the IAM refinements

Measurements for the IAM refinement were integrated with *SAINT* (Bruker, 2019 ▸) using an automatically determined box size. Photon III data sets were integrated using profile fitting for the weak reflections, while the Eiger2 CdTe data sets were integrated without profile fitting (Krause *et al.*, 2020 ▸). Otherwise, settings were kept to identical defaults. Absorption correction and scaling were done in *SADABS* (Krause *et al.*, 2015*a* ▸). The radius for the spherical absorption correction was assumed at half the lowest crystal dimension (Krause *et al.*, 2015*a* ▸). Structures were solved using *SHELXT* (Sheldrick, 2015*b* ▸) and refined against *F* ^2^ using *SHELXL* (Sheldrick, 2015*a* ▸). Initial refinement was done in *ShelXle* (Hübschle *et al.*, 2011 ▸) and afterwards the refinement was automated using a script in Python.

All refinements were carried out against the complete data and against multiplicity normalized data. Normalization was achieved by creating sets of symmetry-equivalent reflections with the same exposure time for both the Eiger2 and Photon III detectors after scaling and absorption correction. The smaller set was included as is, whereas the same number of reflections were drawn randomly from the larger of the two for each symmetry-equivalent reflection/exposure time combination. To quantify any potential bias by the individual random selections, the procedure was repeated 100 times. These sets were subsequently analysed with *XPREP* (Sheldrick, 2015*c* ▸) and individual refinements were conducted using *SHELXL* starting from the refinement against the complete data.

### 2.4. Data processing and refinement for the aspherical refinements

Measurements for the aspherical atom model refinements were integrated with *SAINT* using a variable box size. Subsequently, the measurements were scaled and corrected for absorption using *SADABS*. It has been shown that it is important to check for resolution-dependent errors such as thermal diffuse scattering (Niepötter *et al.*, 2015 ▸), which can be alleviated by applying a resolution-dependent scaling. Accordingly, we used refined scaling factors for all our data sets with the proposed method and corrected the intensity using the following formula, which improved the achieved model significantly:

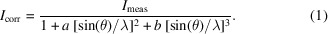

Determined values for *a* and *b* were 0.13/0.76 for the indium/Photon III, −0.08/1.74 for the indium/Eiger2 CdTe and 0.15/1.07 for the silver/Photon III data sets, respectively.

Structures were refined against a pre-solved model using *SHELXL* via the *ShelXle* graphical user interface (Hübschle *et al.*, 2011 ▸). Multipolar refinement was done using the *XD2016* package (Volkov *et al.*, 2016 ▸). Hydrogen atoms bound to carbon atoms in the phenyl ring were placed at a distance of 1.077 Å and the isotropic atomic displacement parameter was set to 1.2 times the *U*_equiv_ value of the bound carbon atom, while the hydrogen atoms in the methyl groups were set at a distance of 1.083 Å and displacement parameters were set to 1.5 times the *U*_equiv_ of the carbon atom. Hirshfeld atom refinement (HAR) was performed using structure factors from periodic projector augmented wave (PAW) density functional theory (DFT) calculations. They employ the SCAN functional (Sun *et al.*, 2015 ▸) in *GPAW* (Mortensen *et al.*, 2005 ▸; Enkovaara *et al.*, 2010 ▸) with grid spacings of 0.16/0.08/0.04 Å (wavefunction/density/fast Fourier transform). At that point the *XHARPy* package (Ruth *et al.*, 2022 ▸) was adopted with all hydrogen atoms refined freely with anisotropic displacement parameters.

## 3. Results

### 3.1. Efficiency of low-energy filtering approaches

For the discussed combination of Excillum MetalJet D2 source with I3 alloy and the Incoatec Helios optics, we still have a significant amount of gallium radiation in the primary beam. Depending on the detector we need to use different measures to get rid of that radiation (Graw *et al.*, 2023 ▸).

For the Photon III detector we used a sheet of 40 µm of palladium to attenuate unwanted radiation. The theoretical attenuation (Hubbell & Seltzer, 2004 ▸) is 99.9% for gallium radiation. The price we pay is an attenuation of 39.4% for indium *K*α as well (Hubbell & Seltzer, 2004 ▸). Compared with an aluminium attenuator, the palladium provides additional filtering above 24.35 keV.

In contrast, the Eiger2 is able to filter out the gallium radiation using the available energy cut-off. As the energy of gallium *K*α (9.2 keV, 1.340 Å) is less than half the energy of indium *K*α (24.1 keV, 0.513 Å) (Deslattes & Kessler, 1985 ▸) the recommended cut-off of half the indium energy is therefore able to suppress the low-energy contamination.

**Figure 2:**
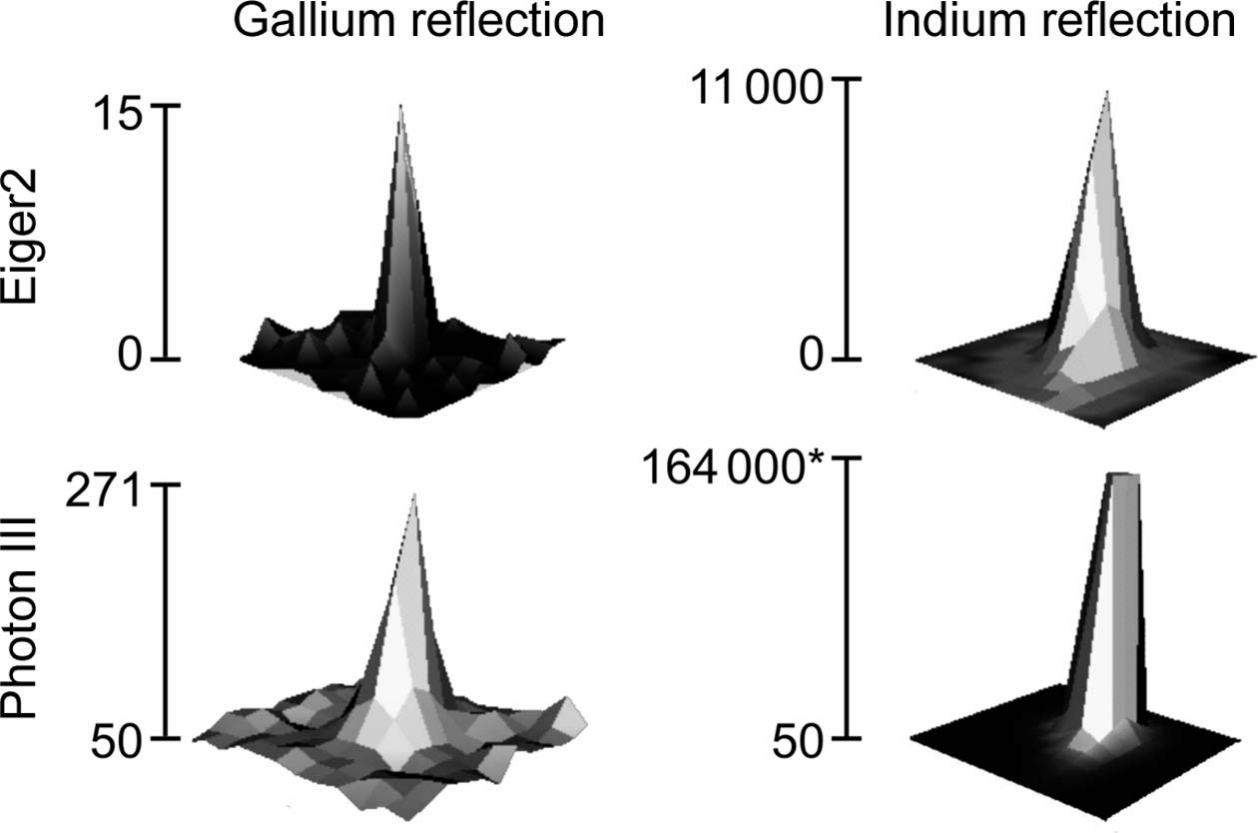
Reflection profiles for 212 measured for 15 s for **1**. The filtering is incomplete for both cases. Note that the height of the Photon III indium reflection (*) is not determined accurately due to overexposure. Height is given in counts.

In order to evaluate to what degree the filtering was actually successful we used two different approaches. Qualitatively, we can evaluate the filtering by examining the strong indium reflection with the Miller index 212 in the data set of **1** and comparing it with its gallium equivalent (Fig. 2 ▸). We see that neither of these setups offers complete suppression of the contamination. Both detectors show a maximum at the position only expected for gallium radiation.

The second, more quantitative, approach used a pseudo-twin refinement of the two sets of reflections to get an impression of the relative strength of the contamination. Cell parameters and orientation and instrument parameters were determined in a first integration with *SAINT*. In a second step, the obtained cell was scaled to match the equivalent gallium reflection positions. With fixed orientation and cell parameters, the relative intensities were determined in a second integration using the twin feature of *SAINT*. After absorption correction and frame-to-frame scaling in *TWINABS* (Sevvana *et al.*, 2019 ▸), the two components were refined as a twin in *SHELXL*. The determined twin partitions were 0.226 (11)% for the Eiger2 and 0.44 (4)% for the Photon III detector. The relative percentages are more indicative than the absolute ones, as effects such as scattering cross sections and absorption have been neglected but should be the same for both detectors. Therefore, we could show a very small residual contamination with a slight advantage of the Eiger2 detector. A higher thickness of the palladium attenuator would have solved the problem, but would have led to a lower overall indium intensity.

### 3.2. Precision of the measured data

The precision was evaluated using the redundancy-independent merge *R*_r.i.m_ (Weiss, 2001 ▸), the precision-indicating merge *R*_p.i.m_ and the average intensity over estimated error *I*/σ. These were evaluated using both the complete data set and the redundancy-equivalent data sets prepared according to the procedure described in Section 2.3. The results are shown in Table 2 ▸.

The data sets were obviously not collected with the same multiplicities. On the one hand, this is due to the larger active area of the Photon III detector. On the other hand, we also included multiple runs with increasing exposure times on the Photon III to make sure all reflections were measured with an exposure time as high as possible, while being limited by the over-exposure limit. The Eiger2 therefore also has a lower number of runs, as the maximum exposure limit is higher for this detector. Obviously, the multiplicity-equivalent data sets have the same multiplicity.

The redundancy-independent merge factor (*R*_r.i.m_) shows the superior performance of the Eiger2 detector for each of the structures studied in this work for both the overall and multiplicity-equivalent data sets.

For the *R*_p.i.m_ and *I*/σ values the Photon III profits from the higher multiplicity. Consequently, three of the four full data sets are superior for the Photon III detector. After making the multiplicity equivalent, the Eiger2 shows superior performance for all data sets.

The relative performance on the precision indicators is basically retained when we compare the performance of the two detectors with indium radiation for the measurement of structure **5** (Table 3 ▸). The Eiger2 data set shows essentially identical performance to the silver data set at a higher collected multiplicity. The data collected on the Photon III detector with the indium MetalJet show inferior quality indicators compared with the other two measurements. Finally, the intensity/data ratio of the Eiger/In combination is superior to both comparison data sets.

### 3.3. Quality indicators of evaluated IAM refinements

As a first possible application we evaluated the performance of the MetalJet using either detector for IAM refinement in *SHELXL* with different quality indicators. Again, we evaluated the performance both for the entire collected data set for each detector and for the data sets which had been reduced to be multiplicity equivalent. In accordance with common practice a weighting scheme was refined.

The results are listed in Table 4 ▸. The crystallographic agreement factor shows a higher agreement for the Eiger2 detector for the evaluated structures derived from the complete reflection sets for **1** and **3**, an improved performance for the Photon III for **2**, and a very similar performance for **4**. The normalized data sets show the superior performance of the Eiger2 for all four structures. The *e*_gross_ value (Meindl & Henn, 2008 ▸) describes the number of non-assigned electrons in the difference electron density. Here the Photon III detector shows a lower number of undescribed electrons for the complete data set of **2** compared with the Eiger2 detector. For all other data sets, including all the normalized data sets, the Eiger2 shows a lower number of undescribed electrons.

The uncertainties in the evaluated bonds were basically identical for the two detectors. This indicates that, despite their different performances, the MetalJet source is suitable for measurements with either detector and the choice does not affect the uncertainties in the determined bond lengths, which is usually the aim of the IAM refinement. In all other quality indicators, the Eiger2 shows superior performance to the Photon III, which can be explained by the use of the palladium attenuator for removing the low-energy gallium contamination, as well as the lower photon efficiency of the evaluated Photon III detector compared with the Eiger2 CdTe.

### 3.4. Comparison of aspherical refinements of the YLID crystal

The electron density around an atom can be mathematically separated into a spherically symmetrical part and the aspherical contributions. The IAM discussed in Section 3.3 approximates the atomic electron density with densities calculated for isolated atoms (Doyle & Turner, 1968 ▸; Prince, 2004 ▸). More sophisticated models can also account for aspherical contributions which are almost exclusively found in the valence density. We measured an YLID crystal to compare the performance of the two detectors on the MetalJet with the performance of an Incoatec IμS 3.0 Ag source for two different approaches to the aspherical description, namely Hirshfeld atom refinement (HAR) (Jayatilaka & Dittrich, 2008 ▸; Capelli *et al.*, 2014 ▸) and multipole refinement (Coppens, 1997 ▸).

#### 3.4.1. Comparison of quality indicators

**Figure 3:**
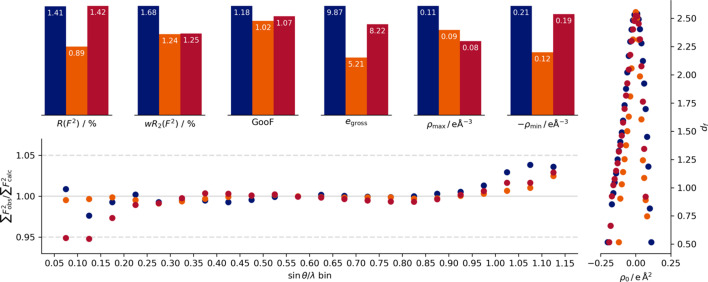
Quality indicators of the different measurements for the refinement against atomic form factors from Hirshfeld partitioning for the MetalJet–Photon III (blue), MetalJet–Eiger2 (orange) and IμS 3.0 Ag–Photon III (red) setups. At the top are the quality indicators, on the right-hand side is a Henn–Meindl plot (Meindl & Henn, 2008 ▸) and at the bottom is a plot produced to mimic *DRKPlot* (Stash, 2007 ▸; Zavodnik *et al.*, 1999 ▸; Zhurov*et al.*, 2008 ▸) output, but not using *DRKPlot* itself.

The crystallographic quality indicators are very similar for both methods (Figs. 3 ▸ and 4 ▸): The performances of the unweighted crystallographic agreement factor *R*(*F* ^2^) and *e*_gross_ follow the *I*/σ indicator of the data collection. Accordingly, we see the superior performance of the agreement with the data collected by the Eiger2 CdTe on the In MetalJet, separated by a significant margin from the IμS Ag measurement using the Photon III. The In MetalJet measurement on the Photon III shows the highest values and number of undescribed electrons *e*_gross_ for both evaluated methodologies. For the unweighted agreement factor, both Photon III measurements yield the same value for the HAR. However, for the multipole refinement the Photon III In measurement exhibits a higher value than the Photon III measurement using Ag *K*α radiation.

The conclusion is less clear on the weighted crystallographic agreement factor *wR*_2_ and the goodness of fit (GOF). For the Ag/Photon III and In/Eiger2 data, the performance of these two indicators is basically identical for the HAR, while the multipole refinement shows a slight advantage for the Ag/Photon III. The In/Photon III data show inferior performance for these indicators as well.

The Photon III measurements show a higher value in the negative difference electron density for both minima, and also an overall shift in the Henn–Meindl plots. The IμS Ag data also show a slightly lower maximum in the difference electron density, whereas the In/Photon III data set again exhibits the most pronounced maxima and minima in the difference electron density.

The In/Photon III measurement shows a significant jump in the quotient of the sum of observed to the sum of fitted intensities ( 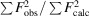 ) between the innermost and second inner resolution shells. The effect is less pronounced in the multipole refinement but still visible. The quotient curve of the Eiger2 is smooth for the Hirshfeld refinement. The resulting refinement of the Ag/Photon III measurement shows under-determined intensities for the two innermost shells. We attribute the difference to the larger number of lower exposure time reflections in the Photon III data set, as the higher exposure times are not available due to overexposure. The Eiger2 does not suffer from overexposure to the same degree.

**Figure 5:**
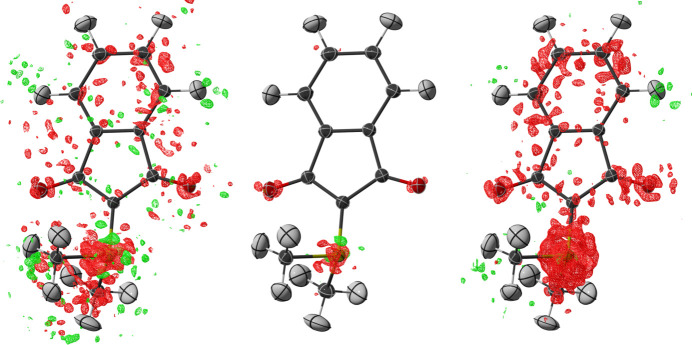
Difference electron densities at isolevels ±0.05 e Å^−3^ for the Hirshfeld atom refinements of **5** for data obtained on (left) the indium/Photon III, (centre) the indium/Eiger2 CdTe and (right) the silver/Photon III setups. Atomic displacement parameters are depicted at the 50% probability level.

The difference electron densities of the Hirshfeld atom refinement (Fig. 5 ▸) and the multipolar model (Fig. S9 in the supporting information) closely follow the Henn–Meindl plots. All refinements show a low level of difference electron density. The indium MetalJet data obtained with the Photon III detector show a noisy overall difference electron density at an isolevel ±0.05 e Å^−3^, with the highest features being located near the heaviest atom, namely the ylid sulfur. At the same time the IμS 3.0 Ag data with the Photon III show a disposition towards a negative difference electron density, which can be explained by the intensity of the inner data matching less accurately, as observed in the *DRKPlot*-type plot. In comparison, the difference electron density obtained by the indium MetalJet with the Eiger2 CdTe is much flatter. The visible features at the same low isolevel (±0.05 e Å^−3^) are limited to the vicinity of the sulfur atom and the oxygen atoms, while also being less strongly expressed at these positions. The resulting difference electron densities of the multipolar refinements are comparable for the three investigations (Fig. S9). In the IμS 3.0 Ag/Photon III data, the increased number of parameters does counteract the overall negative density near the sulfur atom and part, but not all, of the discrepancy is assigned to the density, as investigated in the next section.

#### 3.4.2. Comparison of derived QTAIM properties of the multipole refinement

**Figure 6:**
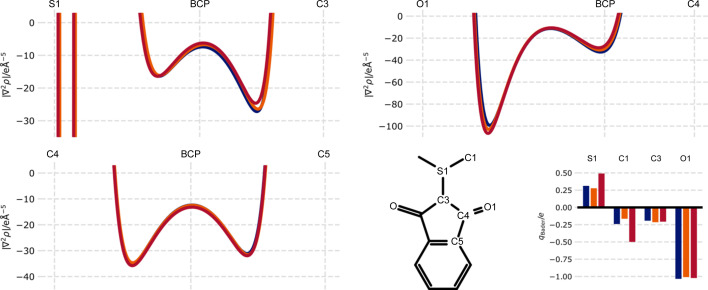
Selected QTAIM properties derived from the multipolar refinement for the MetalJet–Photon III (blue), MetalJet–Eiger2 (orange) and IμS 3.0 Ag–Photon III (red) setups. Depicted are the Laplacians along the bond bath for three different atom pairs (top left, top right, bottom left) with marked atom positions and bond critical point (BCP) position. (Bottom centre) The corresponding labelling of the atoms and (bottom right) the integrated Bader charges for selected atoms.

We now want to compare derived properties from the different measurements. Currently, the usual aim for multipole refinements is to derive a density which can be subsequently analysed with QTAIM. Selected examples of derived properties are depicted in Fig. 6 ▸; a detailed analysis of molecular structure within the QTAIM framework is provided by Graw *et al.* (2023 ▸) on the basis of the experimental charge density derived from the Indium/Eiger2 CdTe data.

Laplacians along bond paths are conserved well between the different measurements and the Laplacian values at the BCPs are basically indistinguishable. In contrast, the integrated Bader charges differ for the sulfur atom of the YLID and the connected methyl groups. We attribute this difference again to the Photon III’s difficulty in measuring the strongest reflections, which in turn significantly affects the density of the heaviest sulfur atom. The effect on the lighter oxygen atoms and the remaining carbon atoms within the structure is negligible. Again, all three measurements yield similar Bader charges.

## 4. Conclusions

We have successfully demonstrated the first application of MetalJet In *K*α radiation to single-crystal X-ray crystallography and evaluated different detectors, the Photon III and the Eiger2 CdTe, for usage on this machine in investigations involving spherical and aspherical models.

Overall, the Eiger2 CdTe proved to be the solution to the specific MetalJet problem of contamination with gallium *K*α radiation. Instead of needing to remove the radiation with an attenuator, which also affects the intensity of the indium radiation used for measurement, the second higher wavelength can be elegantly removed using the available energy cut-off.

Both implementations are suitable for measurements for the purpose of independent-atom model refinement, but the Eiger2 CdTe shows superior performance in quality indicators for the precision of the individual measurements, as well as an improvement in the quality indicators of the crystallographic model. The increase in precision could be demonstrated when the difference in multiplicity was removed.

We also evaluated the performance for a charge-density refinement of the YLID crystal for both detectors, as well as an IμS 3.0 Ag source with a Photon III detector. Again, all three setups provide really good quality models. For this crystal, the In MetalJet with an Eiger2 CdTe detector was able to produce data better described by the evaluated multipole and Hirshfeld atom descriptions. In a direct comparison of the Photon III measurements with the MetalJet and the IμS 3.0 Ag sources, the IμS shows improved performance. Further evaluation in this direction is necessary but was beyond the scope of this publication.

Overall, the MetalJet source used with indium radiation provides an interesting setup, with a potentially higher resolution and fast measurement times for compounds with smaller unit cells. Harder radiation should reduce artefacts resulting from high absorption and extinction. The narrow beam is well suited to small samples of such compounds. The instrument still carries untapped potential in the form of a higher voltage generator. While the current 70 kV high-voltage generator provides a suitable intensity for the investigated measurements, exploration of the current setup with a 160 kV generator still remains to be conducted, with an expected increase in achievable intensity.

## 5. Related literature

For further literature related to the supporting information, see Parsons *et al.* (2013 ▸) and Spek (2003 ▸).

## Supplementary Material

Crystal structure: contains datablock(s) ScCoC_eiger, ScCoC_photon, ScPtSi_eiger, ScPtSi_photon, NaWO4_eiger, NaWO4_photon, LAla_eiger, LAla_photon, Ylid_HAR_Ag_Photon, Ylid_HAR_In_Eiger, Ylid_HAR_In_Photon, Ylid_MM_Ag_Photon, Ylid_MM_In_Eiger, Ylid_MM_In_Photon. DOI: 10.1107/S1600576723007215/nb5354sup1.cif

Structure factors: contains datablock(s) ScCoC_eiger. DOI: 10.1107/S1600576723007215/nb5354ScCoC_eigersup2.hkl

Structure factors: contains datablock(s) ScCoC_photon. DOI: 10.1107/S1600576723007215/nb5354ScCoC_photonsup3.hkl

Structure factors: contains datablock(s) ScPtSi_eiger. DOI: 10.1107/S1600576723007215/nb5354ScPtSi_eigersup4.hkl

Structure factors: contains datablock(s) ScPtSi_photon. DOI: 10.1107/S1600576723007215/nb5354ScPtSi_photonsup5.hkl

Structure factors: contains datablock(s) NaWO4_eiger. DOI: 10.1107/S1600576723007215/nb5354NaWO4_eigersup6.hkl

Structure factors: contains datablock(s) NaWO4_photon. DOI: 10.1107/S1600576723007215/nb5354NaWO4_photonsup7.hkl

Structure factors: contains datablock(s) LAla_eiger. DOI: 10.1107/S1600576723007215/nb5354LAla_eigersup8.hkl

Structure factors: contains datablock(s) LAla_photon. DOI: 10.1107/S1600576723007215/nb5354LAla_photonsup9.hkl

Structure factors: contains datablock(s) Ylid_HAR_Ag_Photon. DOI: 10.1107/S1600576723007215/nb5354Ylid_HAR_Ag_Photonsup10.hkl

Structure factors: contains datablock(s) Ylid_HAR_In_Eiger. DOI: 10.1107/S1600576723007215/nb5354Ylid_HAR_In_Eigersup11.hkl

Structure factors: contains datablock(s) Ylid_HAR_In_Photon. DOI: 10.1107/S1600576723007215/nb5354Ylid_HAR_In_Photonsup12.hkl

Structure factors: contains datablock(s) Ylid_MM_Ag_Photon. DOI: 10.1107/S1600576723007215/nb5354Ylid_MM_Ag_Photonsup13.hkl

Structure factors: contains datablock(s) Ylid_MM_In_Eiger. DOI: 10.1107/S1600576723007215/nb5354Ylid_MM_In_Eigersup14.hkl

Structure factors: contains datablock(s) Ylid_MM_In_Photon. DOI: 10.1107/S1600576723007215/nb5354Ylid_MM_In_Photonsup15.hkl

Additional refinement details. DOI: 10.1107/S1600576723007215/nb5354sup16.pdf

## Figures and Tables

**Figure 4:**
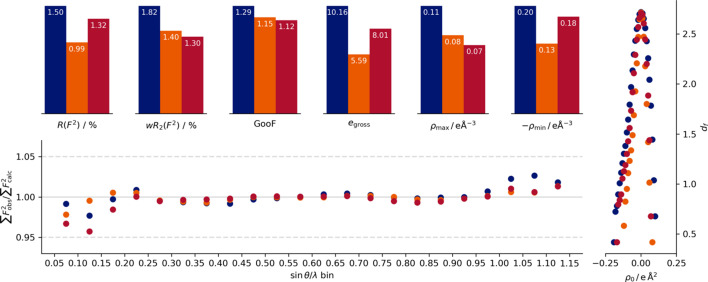
Quality indicators of the different measurements for the refinement against a multipole model for the MetalJet–Photon III (blue), MetalJet–Eiger2 (orange) and IμS 3.0 Ag–Photon III (red) setups. At the top are the quality indicators, on the right-hand side is a Henn–Meindl plot (Meindl & Henn, 2008 ▸) and at the bottom is a plot produced to mimic *DRKPlot* output, but not using *DRKPlot* itself.

**Table 1:** Crystal properties and measurement settings for the comparison measurements Values for μ and μ*r* are given for In *K*α for structures **1** to **4**. Values for structure **5** are given for In *K*α/Ag *K*α.

	Space group	Crystal dimensions (mm)	μ (mm^−1^)	μ*r*	*d*_min_ (Å)	*T* (K)
**1** [ScCoC_4_]_*n*_	*Immm*	0.592 × 0.063 × 0.031	3.9	0.06	0.39	100
**2** [ScPt_9_Si_3_]_*n*_	*C*2/*c*	0.059 × 0.049 × 0.041	64.3	1.29	0.38	100
**3** [Na_2_WO_4_·2H_2_O]_*n*_	*Pbca*	0.208 × 0.157 × 0.086	8.2	0.35	0.36	100
**4** L-Ala	*P*2_1_2_1_2_1_	0.214 × 0.155 × 0.128	0.06	0.00	0.45	150
**5** YLID	*P*2_1_2_1_2_1_	0.395 × 0.387 × 0.312	0.13/0.16	0.03/0.02	0.45	110

**Table 2:** Data descriptors for the evaluated IAM data sets For the individual data sets, the numbers in rows marked ‘Full’ were determined from the overall data, while rows marked ‘Equal’ contain values which were determined from data cut to be multiplicity equivalent. If the determined multiplicity-equivalent values are all equivalent, no sample standard deviation is listed.

			**1**	**2**	**3**	**4**
			[ScCoC_4_]_*n*_	[ScPt_9_Si_3_]_*n*_	[Na_2_WO_4_·2H_2_O]_*n*_	L-Ala
Multiplicity	Full	Eiger2	31.22	7.83	15.31	12.56
		Photon III	60.78	32.63	24.38	31.83
	Equal	Both	25.71	7.76	10.68	7.61

*R*_r.i.m_ (%)	Full	Eiger2	2.35	6.29	3.76	3.75
		Photon III	4.15	9.35	3.97	5.04
	Equal	Eiger2	2.303 (5)	6.3296 (20)	3.502 (5)	3.323 (8)
		Photon III	3.305 (12)	6.98 (2)	4.539 (10)	5.94 (4)

*R*_p.i.m_ (%)	Full	Eiger2	0.39	1.82	0.97	0.90
		Photon III	0.47	1.35	0.69	0.85
	Equal	Eiger2	0.42	1.8397 (17)	1.04	1.099 (5)
		Photon III	0.598 (4)	2.135 (8)	1.339 (3)	1.963 (14)

*I*/σ	Full	Eiger2	117.24	20.03	41.00	41.90
		Photon III	110.13	28.46	44.37	45.61
	Equal	Eiger2	108.063 (13)	19.958 (4)	36.529 (7)	37.73
		Photon III	80.19 (3)	14.830 (9)	28.899 (8)	20.95 (2)

**Table 3:** Quality indicators for the measurement of YLID **5** for aspherical refinement Indicators were evaluated for the full data sets.

Radiation	Detector	Multiplicity	*R*_r.i.m_ (%)	*R*_p.i.m_ (%)	*I*/σ
In	Eiger2 CdTe	29.04	2.48	0.37	101.99
	Photon III	27.99	3.83	0.59	78.14

Ag	Photon III	23.33	2.46	0.39	93.91

**Table 4:** Selected quality indicators for the IAM refinement of structures **1** to **4** For the individual data sets, the values in the left-hand columns were evaluated using the complete data and the values on the right were evaluated from the multiplicity-equivalent data, with the given standard deviation stemming from the 100 draws used for the selection. Bond precisions σ(*d*) were evaluated for the following bond types – **1** Co—C, **2** Pt—Si, **3** W—O and **4** C—C – using our own implemented script.

		**1**		**2**		**3**		**4**	
		[ScCoC_4_]_*n*_		[ScPt_9_Si_3_]_*n*_		[Na_2_WO_4_·2H_2_O]_*n*_		L-Ala	
*R*(*F*) (all data) (%)	Eiger2	0.97	0.930 (5)	3.04	3.029 (3)	1.92	2.079 (6)	3.34	3.221 (2)
	Photon III	1.35	1.352 (15)	2.72	3.85 (3)	2.28	2.361 (6)	3.29	4.53 (4)

*e*_gross_	Eiger2	9.8	5.70 (8)	424.5	419 (7)	143.7	143.0 (5)	10.3	8.45 (3)
	Photon III	10.3	9.52 (8)	395.7	518 (4)	173.2	149.3 (3)	10.8	11.20 (11)

σ(*d*) (mÅ)	Eiger2	0.6	0.6	0.9 (3)	0.9 (3)	0.6	0.6	0.7	0.7
	Photon III	0.7	0.7	0.81 (16)	1.0 (3)	0.7	0.65 (5)	0.6	0.83 (5)
