# Supplementary material for: Indium Kα radiation from a MetalJet X-ray source: comparison of the Eiger2 CdTe and Photon III detectors
Source: J Appl Crystallogr. 2023 Sep 5;56(Pt 5):1322–9. doi: 10.1107/S1600576723007215 (PMC10543668; doi:10.1107/S1600576723007215)
Supplement: Supplementary file 16 [file j-56-01322-sup16.pdf]

JOURNAL OF
APPLIED
CRYSTALLOGRAPHY

Volume 56 (2023)

Supporting information for article:

Indium *K*α radiation from a MetalJet X-ray source: comparison of the Eiger2 CdTe and Photon III detectors

Paul Niklas Ruth, Nico Graw, Tobias Ernemann, Regine Herbst-Irmer and Dietmar Stalke

Crystallographic tables of the non-equivalent complete data sets

Table S1 Crystal and structure refinement data for **1**.

CIF data set identifier	ScCoC_eiger	ScCoC_photon
Radiation type	InK_α	InK_α
Wavelength/Å	0.5134	0.5134
Detector	Dectris Eiger2CdTe	Bruker Photon III
Formula	C_4Sc_3Co	C_4Sc_3Co
$D_{calc.}$/ g cm^{-3}	4.528	4.534
μ/mm^{-1}	3.893	3.899
Estimated T_{min}/T_{max}	0.206/0.889	0.206/0.889
Formula Weight / g mol^{-1}	241.85	241.85
Colour	violet	violet
Shape	needle-shaped	block-shaped
Size/mm^3	0.59×0.06×0.03	0.59×0.06×0.03
T/K	100(2)	100(2)
Crystal System	orthorhombic	orthorhombic
Space Group	*Immm*	*Immm*
a/Å	3.383(2)	3.383(2)
b/Å	4.373(2)	4.370(2)
c/Å	11.991(3)	11.982(3)
V/Å^3	177.39(14)	177.14(14)
Z	2	2
Z'	0.125	0.125
Θ_{min}/°	2.454	2.456
Θ_{max}/°	41.211	41.085
Measured Refl's.	29081	48194
Indep't Refl's	928	923
Refl's I≥2 σ(I)	916	913
R_{int}	0.0249	0.0433
Parameters	18	18
Restraints	0	0
Extinction parameter	0.048(3)	0.071(6)
Largest Peak / e Å^{-3}	0.912	2.892
Deepest Hole / e Å^{-3}	-1.184	-1.835
GooF	1.233	1.165
wR_2 (all data)	0.0260	0.0371
wR_2 [I≥2 σ(I)]	0.0259	0.0371
R_1 (all data)	0.0097	0.0134
R_1 [I≥2 σ(I)]	0.0096	0.0133
Duration of the experiment / h:min:s	20:35:50	33:50:59
Exposure time / s	5 - 20	1 - 20

Table S2 Crystal and structure refinement data for **2**.

**CIF data set identifier**	**ScPtSi_eiger**	**ScPtSi_photon**
Radiation type	InK$_{\alpha}$	InK$_{\alpha}$
Wavelength/Å	0.5134	0.5134
Detector	Dectris Eiger2CdTe	Bruker Photon III
Formula	$Si_3Sc_2Pt_9$	$Si_3Sc_2Pt_9$
$D_{calc.}$/ g cm^{-3}	15.115	15.134
μ/mm^{-1}	64.177	64.254
Estimated T_{min}/T_{max}	0.116/0.178	0.116/0.178
Formula Weight / g mol^{-1}	1930.00	1930.00
Colour	violet	violet
Shape	block-shaped	block-shaped
Size/mm	0.06×0.05×0.04	0.06×0.05×0.04
T/K	100(2)	100(2)
Crystal System	monoclinic	monoclinic
Space Group	$C2/c$	$C2/c$
a/Å	12.976(2)	12.958(3)
b/Å	7.521(2)	7.520(2)
c/Å	9.702(3)	9.711(2)
β/°	116.40(2)	116.47(2)
V/Å^3	848.1(4)	847.1(4)
Z	4	4
Z'	0.5	0.5
Θ_{min}/°	2.330	2.332
Θ_{max}/°	42.536	42.659
Measured Refl's.	64923	272018
Indep't Refl's	8103	8133
Refl's I≥2 σ(I)	7250	7111
R_{int}	0.0633	0.0981
Parameters	67	67
Restraints	0	0
Extinction parameter	0.00155(3)	0.00238(3)
Larest Peak / e Å^{-3}	7.759	6.121
Deepest Hole / e Å^{-3}	-5.386	-5.269
GooF	1.086	1.055
wR_2 (all data)	0.0576	0.0488
wR_2 [I≥2 σ(I)]	0.0565	0.0475
R_1 (all data)	0.0304	0.0272
R_1 [I≥2 σ(I)]	0.0261	0.0221
Duration of the experiment / h:min:s	6:15:00	12:30:24
Exposure time / s	2 - 10	1 -10

Table S3 Crystal and structure refinement data for **3**.

CIF data set identifier	NaWO4_eiger	NaWO4_photon
Radiation type	InK_α	InK_α
Wavelength/Å	0.5134	0.5134
Detector	Dectris Eiger2CdTe	Bruker Photon III
Formula	$O_6H_4Na_2W$	$O_6H_4Na_2W$
$D_{calc.}$/ g cm^{-3}	3.560	3.570
μ/mm^{-1}	8.029	8.052
Estimated T_{min}/T_{max}	0.285/0.544	0.285/0.544
Formula Weight / g mol^{-1}	329.86	329.86
Colour	colourless	colourless
Shape	block-shaped	block-shaped
Size/mm	0.21×0.16×0.09	0.21×0.16×0.09
T/K	100(2)	100(2)
Crystal System	orthorhombic	orthorhombic
Space Group	*Pbca*	*Pbca*
a/Å	8.441(2)	8.434(2)
b/Å	10.569(2)	10.553(2)
c/Å	13.799(3)	13.792(3)
V/Å^3	1231.0(5)	1227.5(5)
Z	8	8
Z'	1	1
Θ_{min}/°	2.132	2.475
Θ_{max}/°	45.573	45.340
Measured Refl's.	220831	341446
Indep't Refl's	13854	13654
Refl's I≥2 σ(I)	12493	12323
R_{int}	0.0363	0.0389
Parameters	99	99
Restraints	7	7
Extinction parameter	0.00253(7)	0.00849(17)
Largest Peak / e Å^{-3}	3.101	4.227
Deepest Hole / e Å^{-3}	-2.919	-4.374
GooF	1.261	1.273
wR_2 (all data)	0.0337	0.0456
wR_2 [I≥2 σ(I)]	0.0332	0.0449
R_1 (all data)	0.0192	0.0228
R_1 [I≥2 σ(I)]	0.0165	0.0200
Duration of the experiment / h:min:s	9:58:00	10:18:24
Exposure time / s	1 - 10	1 -10

Table S4 Crystal and structure refinement data for **4**.

**CIF data set identifier**	**LAla_eiger**	**LAla_photon**
Radiation type	InK_α	InK_α
Wavelength/Å	0.5134	0.5134
Detector	Dectris Eiger2CdTe	Bruker Photon III
Formula	$C_3H_7NO_2$	$C_3H_7NO_2$
$D_{calc.}$/ g cm^{-3}	1.397	1.401
μ/mm^{-1}	0.061	0.061
Estimated T_{min}/T_{max}	0.987/0.992	0.987/0.992
Formula Weight / g mol^{-1}	89.10	89.10
Colour	colourless	colourless
Shape	block-shaped	block-shaped
Size/mm	0.21×0.15×0.13	0.21×0.15×0.13
T/K	100(2)	100(2)
Crystal System	orthorhombic	orthorhombic
Flack Parameter	-0.3(3)	0.3(2)
Space Group	$P2_12_12_1$	$P2_12_12_1$
a/Å	5.789(2)	5.784(2)
b/Å	5.958(2)	5.953(2)
c/Å	12.286(3)	12.272(3)
V/Å^3	423.8(2)	422.6(2)
Z	4	4
Z'	1	1
Θ_{min}/°	2.395	2.398
Θ_{max}/°	34.788	34.821
Measured Refl's.	35004	89949
Indep't Refl's	4857	4857
Refl's I≥2 σ(I)	4437	4569
R_{int}	0.0371	0.0553
Parameters	64	64
Restraints	0	0
Largest Peak / e Å^{-3}	0.560	0.521
Deepest Hole / e Å^{-3}	-0.152	-0.203
GooF	1.081	1.109
wR_2 (all data)	0.0751	0.0792
wR_2 [I≥2 σ(I)]	0.0737	0.0779
R_1 (all data)	0.0334	0.0329
R_1 [I≥2 σ(I)]	0.0301	0.0307
Duration of the experiment /h:min:s	22:22:15	65:44:30
Exposure time / s	3 - 60	1 -60

Table S5 Crystal and structure refinement data for the Hirshfeld atom refinement description of **5**. Flack parameters were determined using Platon (Spek, 2003) with the procedure published by Parsons *et al.* (2013).

**CIF data set identifier**	**Ylid_HAR_In_Eiger**	**Ylid_HAR_In_Photon**	**Ylid_HAR_Ag_Photon**
Radiation type	InK_α	InK_α	AgK_α
Wavelength/Å	0.5134	0.5134	0.56086
Detector	Dectris Eiger2CdTe	Bruker Photon III	Bruker Photon III
Formula	$C_{11}H_{10}O_2S$	$C_{11}H_{10}O_2S$	$C_{11}H_{10}O_2S$
$D_{calc.}$/ g cm^{-3}	1.427	1.432	1.430
μ/mm^{-1}	0.132	0.132	0.163
Estimated T_{min}/T_{max}	0.949/0.960	0.949/0.960	0.938/0.951
Formula Weight / g mol^{-1}	206.25	206.25	206.25
Colour	yellow	yellow	yellow
Shape	spheroid-shaped	spheroid-shaped	spheroid-shaped
Size/mm	0.40×0.39×0.31	0.40×0.39×0.31	0.40×0.39×0.31
T/K	110(2)	110(2)	110(2)
Crystal System	orthorhombic	orthorhombic	orthorhombic
Flack Parameter	0.016(8)	0.024(10)	0.020(7)
Space Group	$P2_12_12_1$	$P2_12_12_1$	$P2_12_12_1$
a/Å	5.860(2)	5.850(2)	5.854(2)
b/Å	8.933(2)	8.924(2)	8.929(2)
c/Å	18.341(3)	18.321(3)	18.328(3)
V/Å^3	960.1(4)	956.5(4)	958.0(4)
Z	4	4	4
Z'	1	1	1
Θ_{min}/°	1.604	1.606	2.002
Θ_{max}/°	34.834	34.793	38.57
Measured Refl's.	178941	171281	142991
Indep't Refl's	11000	10873	10713
Refl's I≥2 σ(I)	10109	10084	9920
R_{int}	0.024	0.0375	0.0237
Parameters	217	217	217
Largest Peak / e Å^{-3}	0.0886	0.1134	0.0768
Deepest Hole / e Å^{-3}	-0.1209	-0.2098	-0.1940
GooF	1.02	1.185	1.07
wR_2 (all data)	0.0124	0.0168	0.0125
wR_2 [I≥2 σ(I)]	0.0124	0.0167	0.0124
R_1 (all data)	0.009	0.0134	0.0111
R_1 [I≥2 σ(I)]	0.0079	0.0116	0.0095
Duration of the experiment / h:min:s	50:43:50	47:40:12	37:49:18
Exposure time / s	5 - 60	1 - 90	1 - 60

Table S6 Crystal and structure refinement data for the Hansen-Coppens-Steward Multipole refinement description of **5**. Flack parameters were determined using Platon (Spek, 2003) with the procedure published by Parsons *et al.* (2013).

**CIF data set identifier**	**Ylid_MM_In_Eiger**	**Ylid_MM_In_Photon**	**Ylid_MM_Ag_Photon**
Radiation type	InK_α	InK_α	AgK_α
Wavelength/Å	0.5134	0.5134	0.56086
Detector	Dectris Eiger2CdTe	Bruker Photon III	Bruker Photon III
Formula	$C_{11}H_{10}O_2S$	$C_{11}H_{10}O_2S$	$C_{11}H_{10}O_2S$
$D_{calc.}$/ g cm^{-3}	1.427	1.432	1.430
μ/mm^{-1}	0.132	0.132	0.163
Formula Weight / g mol^{-1}	206.25	206.25	206.25
Colour	yellow	yellow	yellow
Shape	spheroid-shaped	spheroid-shaped	spheroid-shaped
Size/mm	0.40×0.39×0.31	0.40×0.39×0.31	0.40×0.39×0.31
T/K	110(2)	110(2)	110(2)
Crystal System	orthorhombic	orthorhombic	orthorhombic
Flack Parameter	0.015(6)	0.033(8)	0.016(6)
Space Group	$P2_12_12_1$	$P2_12_12_1$	$P2_12_12_1$
a/Å	5.860(2)	5.850(2)	5.854(2)
b/Å	8.933(2)	8.924(2)	8.929(2)
c/Å	18.341(3)	18.321(3)	18.328(3)
V/Å^3	960.1(4)	956.5(4)	958.0(4)
Z	4	4	4
Z'	1	1	1
Θ_{min}/°	1.604	1.606	2.002
Θ_{max}/°	34.834	34.793	38.57
Measured Refl's.	178941	171281	142991
Indep't Refl's	11000	10873	10713
Refl's I≥2 σ(I)	10109	10084	9920
R_{int}	0.024	0.0375	0.0237
Parameters	250	250	250
Largest Peak / e Å^{-3}	0.068	0.112	0.089
Deepest Hole / e Å^{-3}	-0.113	-0.176	-0.18
GooF	1.15	1.288	1.115
wR_2 (all data)	0.014	0.018	0.013
R_1 (all data)	0.01	0.014	0.011
R_1 [I≥2 σ(I)]	0.01	0.014	0.011

S1. Resolution-dependent quality indicators for the raw data of the IAM refinements.

S1.1. Sc3CoC4

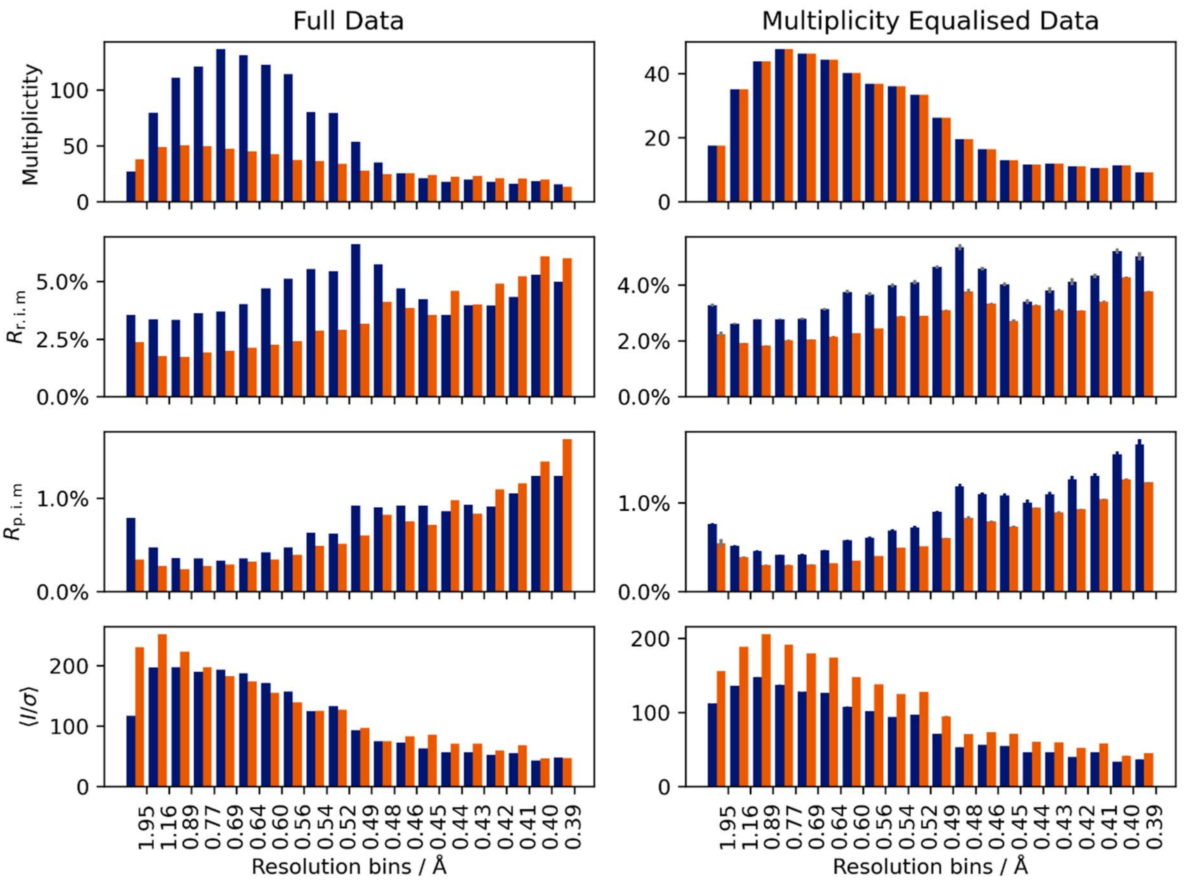

Figure S1 Quality indicators for the raw data of **1**, which were calculated with XPREP in resolution bins. Left: Indicators for the complete collected data. Right: Indicators for the Multiplicity equalised data. The grey indicators indicate the standard deviation of 100 draws from the complete data, but are often below the visible range for the plotted data.

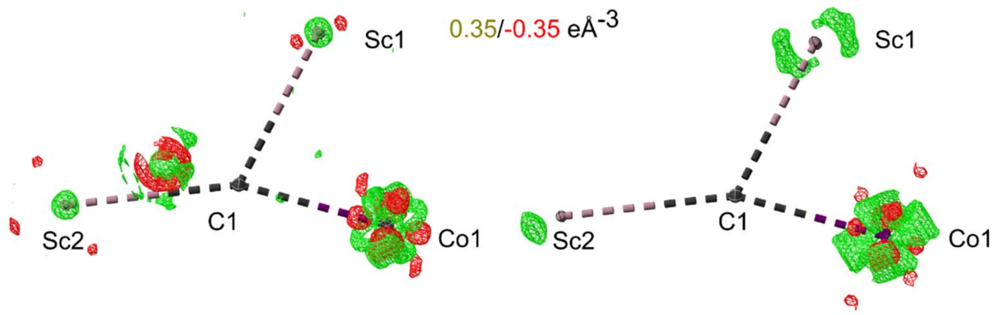

Figure S2 Difference electron density plots for the complete data of **1** for the Photon III detector (left) and the Eiger2 (right). ADPs are depicted at the 50% probability level.

S1.2. ScPtSi

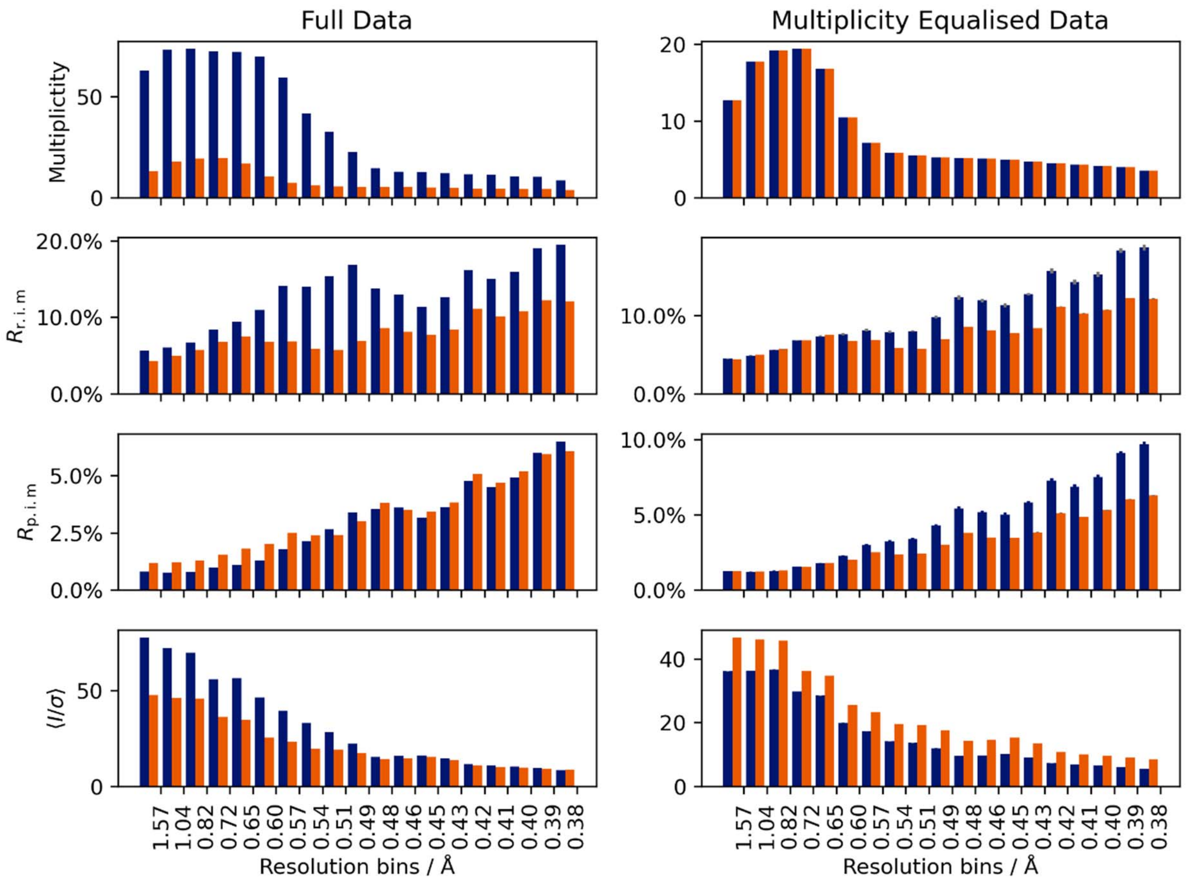

Figure S3 Quality indicators for the raw data of **2**, which were calculated with XPREP in resolution bins. Left: Indicators for the complete collected data. Right: Indicators for the Multiplicity equalised data. The grey indicators indicate the standard deviation of 100 draws from the complete data, but are often below the visible range for the plotted data.

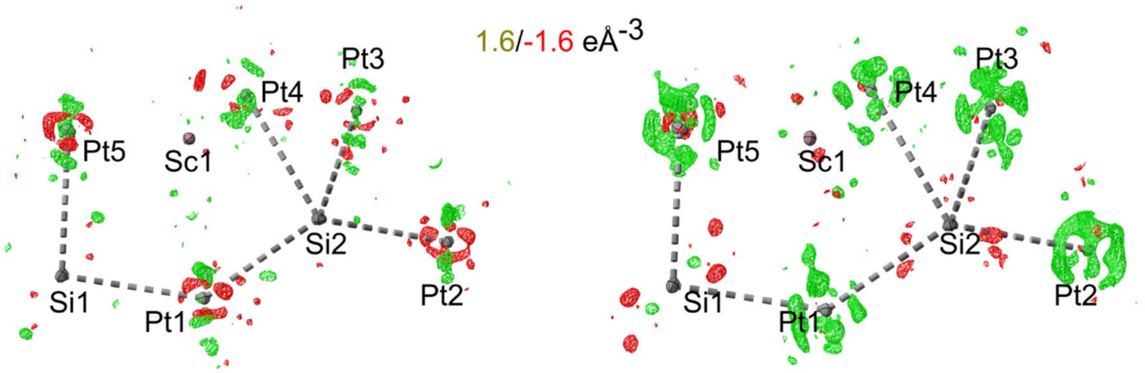

Figure S4 Difference electron density plots for the complete data of **2** for the Photon III detector (left) and the Eiger2 (right). ADPs are depicted at the 50% probability level.

S1.3. NaWO4

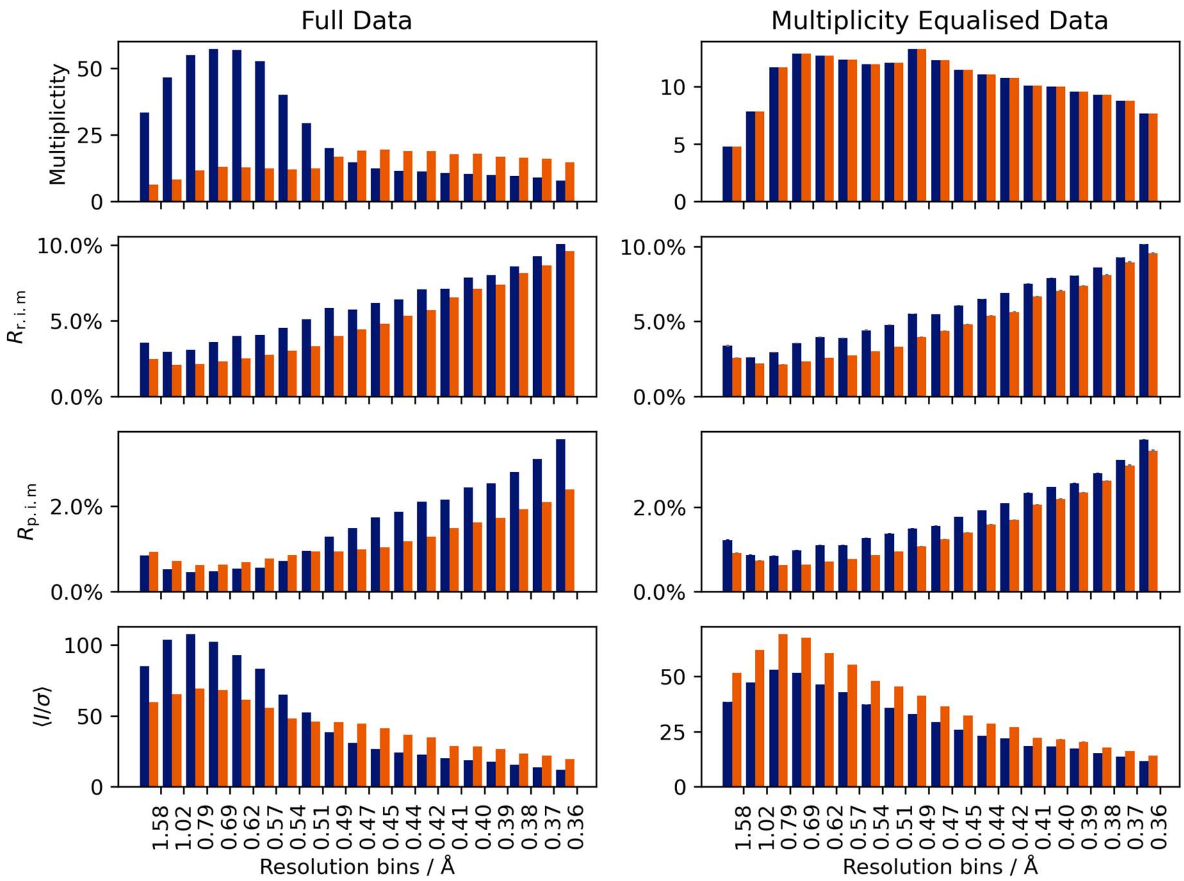

Figure S5 Quality indicators for the raw data of **3**, which were calculated with XPREP in resolution bins. Left: Indicators for the complete collected data. Right: Indicators for the Multiplicity equalised data. The grey indicators indicate the standard deviation of 100 draws from the complete data, but are often below the visible range for the plotted data.

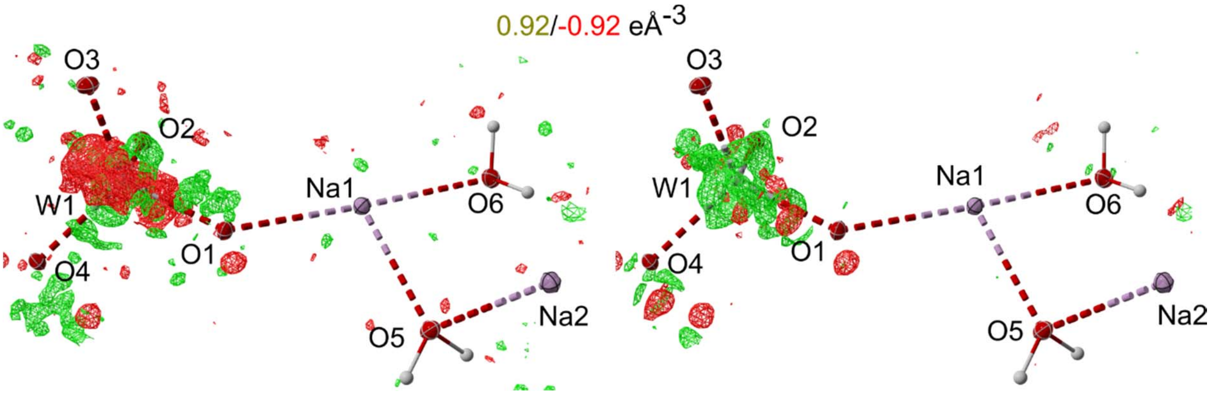

Figure S6 Difference electron density plots for the complete data of **3** for the Photon III detector (left) and the Eiger2 (right). ADPs are depicted at the 50% probability level.

S1.4. LAla

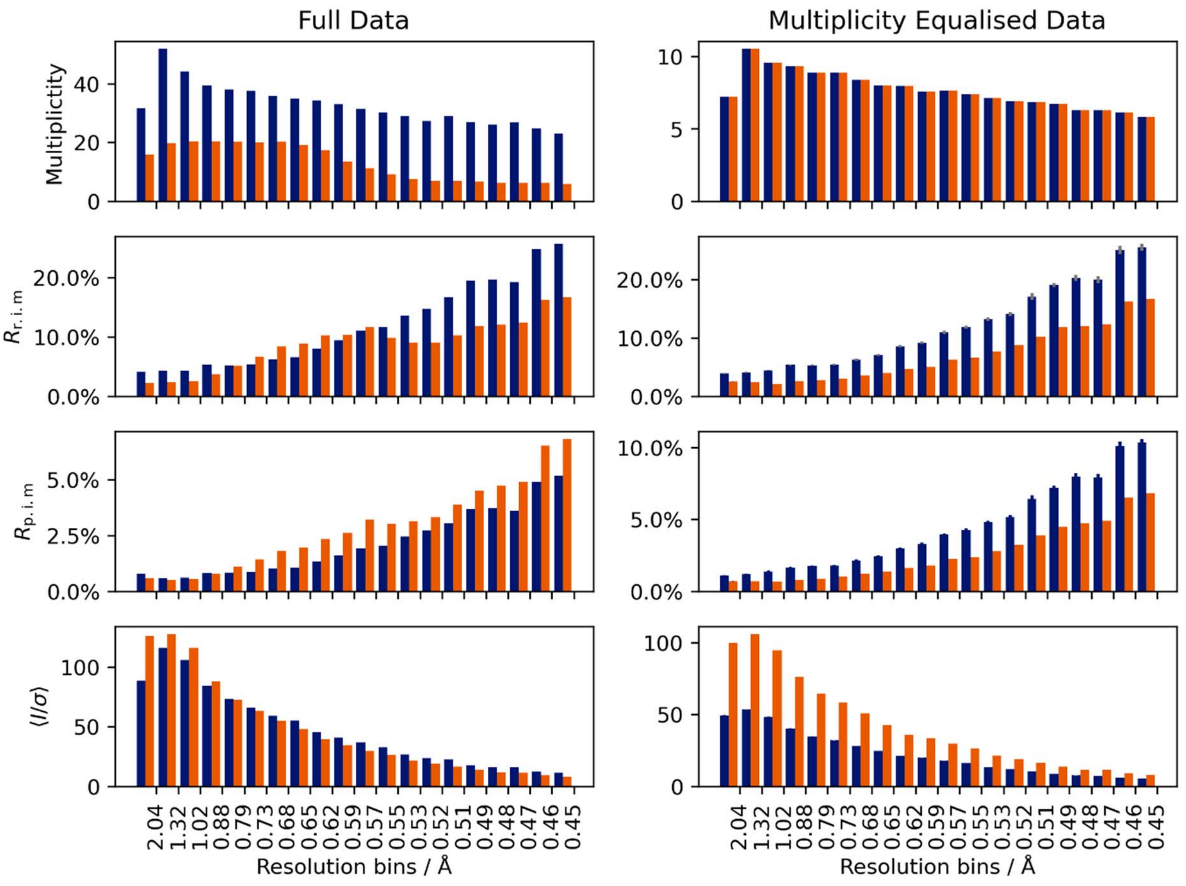

Figure S7 Quality indicators for the raw data of **4**, which were calculated with XPREP in resolution bins. Left: Indicators for the complete collected data. Right: Indicators for the Multiplicity equalised data. The grey indicators indicate the standard deviation of 100 draws from the complete data, but are often below the visible range for the plotted data.

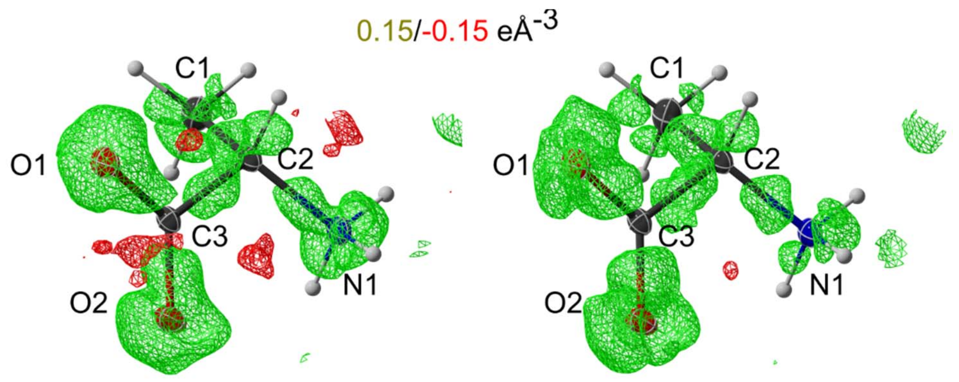

Figure S8 Difference electron density plots for the complete data of **4** for the Photon III detector (left) and the Eiger2 (right). ADPs are depicted at the 50% probability level.

S1.5. Calculation of e_{gross} values for IAM structures

e_{gross} values were calculated using difference electron densities obtained from the fcf6 files of SHELXL with cctbx using the grids listed in Table S1 by summing the absolute values of all grid points and dividing it by 2.

Table S7 Grid sizes used for the calculation of e_{gross}. All grids were obtained with a map factor of 1/6

1	2	3	4
(54, 72, 192)	(216, 120, 160)	(144, 180, 240)	(80, 80, 180)

S2. Aspherical atom refinements

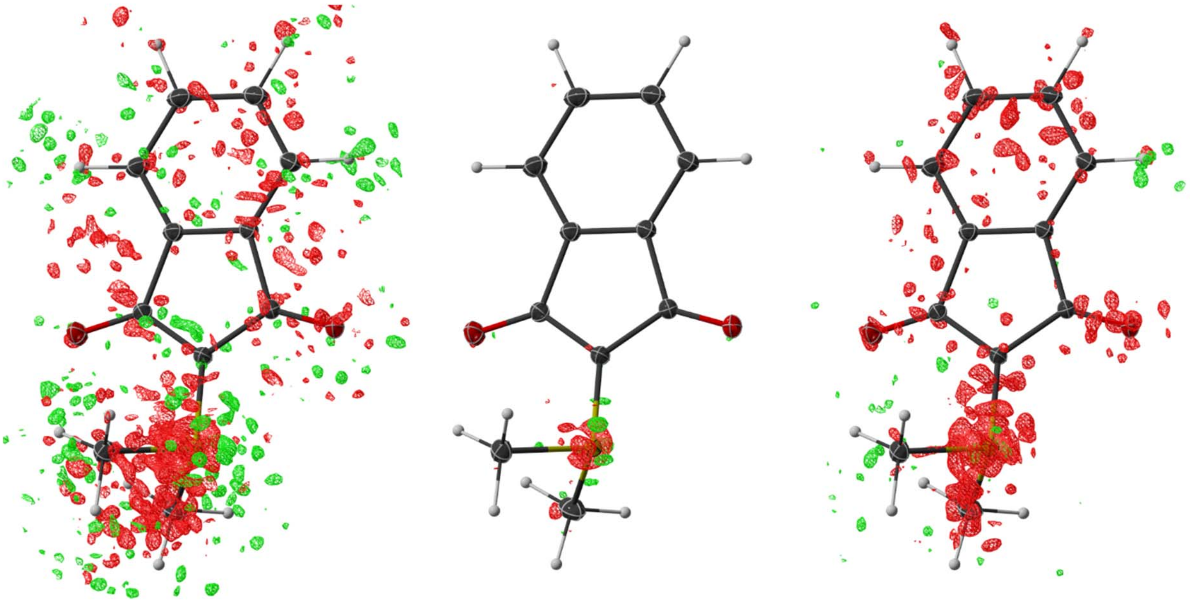

Figure S9 Difference electron densities at isolevels -0.05/0.05 for the Multipole refinements of **5** for the data obtained on the indium/Photon III (left), indium/Eiger2 CdTe (centre) and silver/Photon III (right) setups. Atomic displacement parameters are depicted at the 50 % probability level.

References

Parsons, S., Flack, H. D. & Wagner, T. (2013). *Acta Cryst.* B**69**, 249-259, doi: 10.1107/S2052519213010014.

Spek, A. L. (2003). *J. Appl. Cryst.* **36**, 7-13, doi: 10.1107/S0021889802022112.
